# Research on Distributed 5G Signal Coverage Detection Algorithm Based on PSO-BP-Kriging

**DOI:** 10.3390/s18124390

**Published:** 2018-12-11

**Authors:** Tingli Xiang, Hongjun Wang

**Affiliations:** Institute of Electronic Countermeasure, National University of Defense Technology, Hefei 230037, China; xiangtl1994@gmail.com

**Keywords:** 5G mobile communication network, distributed sensor network, neural network, interpolation, PSO-BP-Kriging

## Abstract

In order to overcome the limitations of traditional road test methods in 5G mobile communication network signal coverage detection, a signal coverage detection algorithm based on distributed sensor network for 5G mobile communication network is proposed. First, the received signal strength of the communication base station is collected and pre-processed by randomly deploying distributed sensor nodes. Then, the neural network objective function is modified by using the variogram function, and the initial weight coefficient of the neural network is optimized by using the improved particle swarm optimization algorithm. Next, the trained network model is used to interpolate the perceptual blind zone. Finally, the sensor node sampling data and the interpolation estimation result are combined to generate an effective coverage of the 5G mobile communication network signal. Simulation results indicate that the proposed algorithm can detect the real situation of 5G mobile communication network signal coverage better than other algorithms, and has certain feasibility and application prospects.

## 1. Introduction

With the popularity of data services and smart terminals, 4G networks fails to satisfy people’s requirements in terms of capacity, speed, bearer, and spectrum. Compared with 4G technology, 5G technology has greatly improved data transmission rates and spectrum resource utilization, and the user experience, wireless signal coverage, and signal transmission stability have also been significantly improved. It has the characteristics of low latency, low power consumption, security, stability, and reliability [1,2]. Since 5G mobile communication network will realize further innovation and integration in the fields of wireless, terminal, service and application scenarios, it has become an inevitable trend and research hotspot in the field of communication.

The future 5G network will be characterized by high automation, intelligence, flexibility, high efficiency, and stability. In order to better cope with the challenges brought by the development of 5G networks, academia and industry around the world have launched in-depth research on 5G mobile communication technologies, including the 5th Generation Non-Orthogonal Waveforms for Asynchronous Signaling (5GNOW) [3], Mobile and Wireless Communications Enablers for the Twenty-Twenty Information Society (METIS) project [4], 5G Public-Private Partnership (5G PPP) project, China’s IMT-2020 (5G) promotion group [5], South Korea’s 5G Forum (5G Forum) and Japan’s 5G research group “2020 and Beyond Ad Hoc” [5,6,7]. Up to now, the overall vision and performance requirements of the future 5G network have reached a global consensus, including higher data traffic and user experience rate, massive terminal connections, lower latency, higher reliability, and so on [5,6,7,8,9,10,11].

It is expected that the data traffic of 5G network will increase 1000 times more than that of the 4G network in the future, and the user experience rate will also increase 10 to 100 times. In order to meet these requirements, 5G will present new breakthroughs in several key technologies, including wireless transmission technology and ultra-dense heterogeneous network technology [12,13,14]. On the one hand, advanced wireless transmission techniques can be utilized, or spectrum bandwidth can be increased to increase spectrum resource utilization. On the other hand, improving spatial multiplexing by cryptographic cell deployment is still the most effective way to increase the capacity of wireless communication systems. Traditional wireless communication systems usually use cell splitting to reduce cell radius. However, with the further reduction of cell coverage, cell splitting will be difficult to carry out. It is necessary to deploy small low-power base stations intensively in indoor and outdoor hot spots to form a super-dense heterogeneous network architecture. Due to the lower transmit power and smaller cell radius of 5G mobile communication networks, high-precision detection of 5G mobile communication network base station signal coverage has become one of the current research hotspots and cutting-edge technologies.

At present, the more mainstream scheme of mobile communication network coverage detection is to use vehicle-borne or hand-held test terminals and frequency sweepers to perform road tests [15,16]. The change of the direction of the smart antenna beam makes the traditional road test method unsuitable for the 5G mobile communication network. The current 5G mobile communication network is in the critical stage of testing and trial, and the signal coverage detection should be anytime, anywhere, and repeatable. Traditional road test methods are time-consuming and laborious, and they are not feasible in varied wild, especially harsh environments, failing to meet the technical requirements of the current testing phase. In addition, although the 5G network adopts a series of technologies, such as soft-defined cloud architecture, network virtualization and slicing, and establishes computing and storage capabilities on the base station through edge computing to achieve low latency of network services, the base station is physically present, and provides access and information interaction for users through wireless transmission technology. Therefore, it is necessary to come up with a method that can test 5G wireless coverage at any time, repeatably and in real time. This paper proposes a research scheme for signal coverage detection through distributed sensor networks based on ad hoc network technology, and the main contributions of our work are as follows:

1. Distributed sensor nodes are randomly deployed to collect the received signal strength indicator (RSSI) of 5G communication base station, and the collected data are pre-processed by Gaussian filtering, which reduces the influence of error on the performance of the algorithm.
2. The Delaunay triangulation algorithm is used to mesh the target area, and the selection of interpolation points is realized.
3. An improved hybrid interpolation algorithm is proposed to estimate the RSSI value of the interpolation point. The objective function of backpropagation (BP) neural network is modified by the variogram of Kriging interpolation and improved particle swarm optimization (PSO) algorithm, which overcomes the overly smooth spatial expression of traditional Kriging interpolation and local convergence of BP neural network interpolation.
4. The data collected by the sensor node and the data estimated by the interpolation point are processed comprehensively, then the coverage area situation of the 5G mobile communication network is generated, realizing the reproducible real-time detection of the wireless network coverage.

The rest of the paper is organized as follows: We first introduce the relevant research background of the work in Section 2, and analyze the related literature cited. Then, in Section 3, the basic theoretical knowledge of the algorithm is described. The architecture and specific steps of the algorithm are described in Section 4. In Section 5, the performance of the algorithm is evaluated and the coverage situation of the 5G mobile communication network is generated. Finally, we summarize our work in Section 6.

## 2. Related Work

The proposed scheme collects the RSSI values through the wireless sensor nodes deployed in the target area of the 5G mobile communication network, and then comprehensively processes the data collected by all the sensor nodes to generate the network coverage situation of the target area, thereby realizing all-round automated sensing and satisfying 5G mobile communication network coverage situation detection special requirements, such as real-time field reproducible detection [17]. By means of UAV carrying and other methods, the wireless sensor node can be placed in the area to be tested, especially in the area that is difficult to reach by traditional road test.

Since the data perceived by the distributed sensor node is only the RSSI value at the location of the node, the final coverage of the 5G mobile communication network in the entire area is obtained. Therefore, it is necessary to estimate the RSSI value for other areas that the sensor node cannot perceive.

At present, there are mainly two methods for RSSI value estimation: signal propagation model estimation method and interpolation estimation method [18]. The signal propagation model estimation method is based on the distribution trend of the RSSI values data collected by the sensor nodes, and the appropriate loss model is selected for estimation [19]. The complexity of the algorithm is low, but the existing models usually cannot accurately match the complex and varied geographical environment of the target area, resulting in low precision. So far, no mature model suitable for 5G networks has been developed. However, the interpolation estimation method based on the feature attributes of nodes in the neighborhood is relatively feasible and has high precision [20]. Commonly used interpolation estimation methods include inverse distance weighted interpolation, Newton interpolation, Kriging interpolation and so on. In [21], the Newton polynomial interpolation method is used instead of the linear interpolation method to estimate the RSSI values, which improves the interpolation precision. However, due to the introduction of the polynomial interpolation function, the computational complexity increases. The inverse distance weighted interpolation method used in [22] has higher precision when the interpolation points are more dispersed. However, since only the positional relationship between nodes is considered, the spatial correlation is poor, and the calculation amount is also large. In [23,24,25], based on the spatial correlation of the RSSI values received by the sensor node, Kriging interpolation method is used to estimate the RSSI values of the perceived blind zone. However, the smoothing effect of the Kriging interpolation tends to obscure important information in areas with sharp changes in spatial data, resulting in inaccurate interpolation expression in this region [26].

In recent years, timing analysis, stochastic simulation, artificial intelligence, and many other methods have been used to overcome the shortcomings of the Kriging interpolation method. Among them, artificial neural networks have strong capabilities in multi-attribute data classification and pattern recognition, and are widely used in many fields such as signal processing [27,28]. Chagas et al. successfully applied neural network technology to positioning problems based on RSSI value estimation in [29,30]. Jia et al. found that Kriging interpolation can better reflect the spatial distribution characteristics of the target region, but the accuracy of neural network interpolation is higher [31]. In [32], an improved model using BP neural network technology instead of Kriging global model is proposed, which is further extended by linear weighted aggregation method to improve the modeling accuracy. Katsuaki et al. proposed a neural Kriging interpolation method, which reproduces the spatial characteristics of regionalized variables and improves the interpolation accuracy to some extent in [33]. However, due to the local convergence of the neural network, the interpolation accuracy of the above algorithm needs to be further improved. In [34,35,36,37,38], the PSO algorithm is used to optimize the weight and threshold of BP neural network, and the PSO-BP model with higher precision and faster convergence rate is obtained. The validity of the model is verified in practical applications.

In order to overcome the shortcomings of the above algorithm application in 5G mobile communication network coverage detection, this paper proposes an improved hybrid interpolation optimization algorithm. Through the correction of the objective function of BP neural network, the algorithm improves the credibility and accuracy of 5G mobile communication network coverage detection.

## 3. Algorithm Description

### 3.1. The Principle of Kriging Interpolation

Kriging interpolation is a linear unbiased estimation method for studying spatial variability and interpolation, which is commonly used in grid statistics in the field of geological survey [39,40]. In the context of this paper, the principle is to estimate the RSSI value of the interpolation point by using the RSSI value received by the sensor nodes in the domain.

Set the RSSI value of the interpolation point to $R(x_{0})$, the RSSI values collected by *m* sensor nodes in the neighborhood are $R(x_{i})(i=1,2,\cdots ,m)$. Then, the estimation formula of Kriging interpolation is defined in Equation (1):

(1) $$R(x_{0})=\sum_{i=1}^{m}\lambda_{i}R(x_{i}),$$

where $\lambda_{i}$ represents the weight of $R(x_{i})$ used for RSSI value estimation in the neighborhood. In order to ensure an unbiased estimation, there is $\sum_{i=1}^{m}\lambda_{i}=1$, and $R(x_{i})$ satisfies the second-order smoothness, thus obtaining

(2) $$\left\{ \begin{array}{l} E[R(x_{i})-R(x_{j})]=0\\ Var[R(x_{i})-R(x_{j})]=E\{[R(x_{i})-R(x_{j}){]}^{2}\} \end{array}\right..$$

To make $R^{*}(x_{0})$ an unbiased estimation of $R(x_{0})$, the estimated variance of $x_{0}$ is required to be the smallest:

(3) $$Var_{\min}(x_{0})=Var[R(x_{0})-R^{*}(x_{0})]=E\{{\left[R(x_{0})-R^{*}(x_{0})\right]}^{2}\}.$$

By introducing the Lagrange multiplier μ to calculate the conditional extremum, it can be expressed as

(4) $$\frac{\partial }{\partial \lambda_{i}}E{\left\{[R(x_{0})-R^{*}(x_{0})\right]}^{2}-2\mu \sum_{i=1}^{m}\lambda_{i}\}=0,$$

where i=1,2,⋯,m, the following Kriging linear equations can be obtained by derivation:

(5) $$\left\{ \begin{array}{l} \sum_{i=1}^{m}\lambda_{i}\gamma (x_{i}-x_{j})+\mu =\gamma (x_{0}-x_{j})\\ \sum_{i=1}^{m}\lambda_{i}=1\;\;\;\;\;\;\;\;\;\;\;\;\;\;\;j=1,2,\cdots ,m \end{array}\right.,$$

where $\gamma (x_{i}-x_{j})=\frac{1}{2}E{\left[R(x_{i})-R(x_{j})\right]}^{2}$ represents the value of variogram between $x_{i}$ and $x_{j}$. Solving Equation (5) gives the weight, $\lambda_{i}$.

The core of the Kriging interpolation is to determine the law of the change of the research object (a variable) with the spatial position according to the feature attributes of the sample point, so as to estimate the attribute value of the interpolation point. This law is the variogram. The variogram is proposed to describe the spatial characteristics of the regionalized variables. The value of variogram can be calculated by the following equation:

(6) $$\gamma (h)=\frac{1}{2N(h)}\sum_{i=1}^{N(h)}{\left[R(x_{i})-R(x_{i}+h)\right]}^{2},$$

where *h* represents the separation distance of a pair of sampling points, and N(h) represents the number of points in all sampling points separated by *h*. The variogram curve γ(h) can be fitted by calculating the value of variogram of different separation distances by Equation (6). From this curve, the value of the variogram between the sample point attribute and the interpolation point attribute in the neighborhood can be obtained, and the Lagrange multiplier μ and the weight $\lambda_{i}$ can be obtained by substituting the value of variogram into Equation (5).

Usually, the existing variogram model is used to fit the curve of variogram by least square method [23]. Based on this, the spatial distribution expressed by the Kriging interpolation is smooth.

### 3.2. The Principle of BP Neural Network Interpolation

BP neural network is a multilayer feedforward neural network based on error back propagation algorithm. Numerous studies have shown that a three-layer BP neural network with sufficient nodes in the hidden layer has the ability to simulate any complex nonlinear mapping [41].

Suppose there are *P* samples, each sample has *N* input components and *M* output components for network training. Calculate the node output by using the node function Equation (7):

(7) $$u_{i}^{k}=f(\sum_{j=1}^{l_{k-1}}w_{i,j}^{k,k-1}u_{j}^{k-1}-\theta_{i}),$$

where $u_{i}^{k}$ is the node output; $w_{i,j}^{k,k-1}$ is the input weight; $\theta_{i}$ is the node threshold; f is the output function, usually taking the Sigmoid function: f(x)=1/(1+exp(−x)).

Calculate the output error by using the objective function F:

(8) $$F=\frac{1}{2}E[\sum_{p=1}^{N}\sum_{j=1}^{M}{\left(y_{j,p}-o_{j,p}\right)}^{2}],$$

where $o_{j,p}$ represents the network output, and $y_{j,p}$ represents the desired output.

When F is less than the set error ε, the network training ends. Interpolation estimation of unknown point attributes can be performed by using the trained network. Although the accuracy of the results estimated by the neural network interpolation method is high, the spatial correlation structure cannot be guaranteed.

## 4. 5G Mobile Communication Network Coverage Detection Algorithm

**Figure 1:**
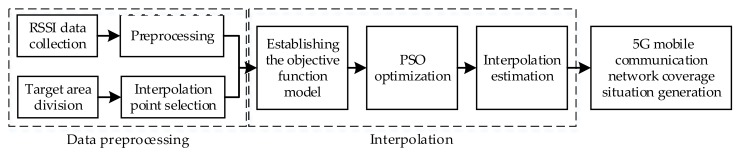
5G mobile communication network coverage detection technology architecture.

Randomly deployed distributed sensor nodes have a certain number of perceived blind zones. The 5G mobile communication network coverage detection technology proposed in this paper uses the hybrid interpolation optimization algorithm to realize the network coverage detection for the perceived blind zone. The algorithm architecture is shown in Figure 1.

The algorithm is mainly composed of three modules: data preprocessing, hybrid interpolation estimation, and 5G mobile communication network coverage situation generation. Among them, data preprocessing mainly completes data collection and processing and target selection. Hybrid interpolation estimation mainly completes the establishment of objective function model, particle swarm optimization, and interpolation estimation. 5G mobile communication network coverage situation generation combines pre-acquired RSSI data and interpolation estimation results to generate equal signal strength lines, then network coverage situation of 5G mobile communication network target area is obtained.

### 4.1. Data Preprocessing

First, to ensure the accuracy of the collected data, it is necessary to filter out small probability interference items in the sample. Since the RSSI values of multiple independent repetitive acquisitions obey the Gaussian distribution, Gaussian filtering can be used to filter out those small probability interference terms [42]. Then, the RSSI values in the range of f(x)≥0.6 (empirical value) in the probability density function Equation (9) are selected, and the mean value is obtained as the sample data required after the preprocessing.

(9) $$f(x)=\frac{1}{\sqrt{2\pi }\sigma }\exp[-\frac{{\left(x-\mu \right)}^{2}}{2\sigma^{2}}],$$

(10) $$\sigma^{2}=\frac{1}{n-1}\sum_{m=1}^{n}{\left({\mathrm{RSSI}}_{m}-\mu \right)}^{2},$$

(11) $$\mu =\frac{1}{n}\sum_{m=1}^{n}{\mathrm{RSSI}}_{m},$$

where ${\mathrm{RSSI}}_{m}$ represents the *m*th acquisition result, *n* represents the number of acquisitions, μ is the mean, and $\sigma^{2}$ is the variance.

Then, the pre-processed sample data are used to divide the target area and select the interpolation points. Delaunay meshing scheme divides the target area into several closed triangles, the sensor node position being the triangle mesh vertex. An interpolation point can be selected in each grid [43]. Due to the limited space, the paper only provides a brief introduction here.

### 4.2. Hybrid Interpolation Optimization Algorithm

#### 4.2.1. Objective Function Establishment

The spatial correlation of the Kriging interpolation mentioned above is good, but the expression is too smooth. The accuracy of the neural network interpolation is high, but the spatial structure is weak. To overcome the shortcomings of the two methods, the objective function is established as follows:

(12) $$\begin{array}{l} F=\frac{1}{2}\left\{\frac{1}{n}\sum_{i=1}^{n}{\left[\frac{y_{i}-o_{i}}{\bar{y}}\right]}^{2}+\frac{1}{N(h_{k})}\sum_{k=1}^{N(h_{k})}{\left[\frac{\gamma (h_{k})-\gamma^{*}(h_{k})}{\bar{\gamma }}\right]}^{2}\right\}+\\ \frac{1}{2}\left\{\frac{1}{n_{1}}\sum_{i=1}^{n}{\left[\max(\frac{o_{i}-o_{\max}}{o_{\max}-o_{\min}},0)\right]}^{2}+\frac{1}{n_{2}}\sum_{i=1}^{n}{\left[\min(\frac{o_{i}-o_{\min}}{o_{\max}-o_{\min}},0)\right]}^{2}\right\} \end{array},$$

where $\gamma (h_{k})$ represents the value of variogram of the sample data; $\gamma^{*}(h_{k})$ represents the value of variogram calculated by the network output; $h_{k}$ is the separation distance of group *k* sensor node pairs; $N(h_{k})$ is the number of points of all sensor nodes separated by $h_{k}$; $\bar{y}$ and $\bar{\gamma }$ are the average values of $y_{i}$ and $\gamma (h_{k})$, respectively; $o_{\max}$ and $o_{\min}$ are the maximum and minimum values of the estimated values, respectively; $n_{1}$ and $n_{2}$ are the number of nodes whose network output is larger than $o_{\max}$ and smaller than $o_{\min}$, respectively.

As a new learning standard, this function contains the error of the variogram and the estimated value, which can effectively improve the interpolation expression of the neural network.

#### 4.2.2. Improved Particle Swarm Optimization Algorithm for BP Neural Network

Although the BP neural network error back propagation algorithm tends to converge to a small network, it is likely to fall into the local minimum under the condition of training complex data distribution patterns. Particle swarm optimization has the characteristics of easy implementation, high efficiency, and intelligence [44]. By introducing the neural network objective function into the particle swarm fitness function, the initial weight coefficient can be optimized. However, the standard particle swarm optimization algorithm is also likely to fall into local optimum. In order to improve the effectiveness of the algorithm, we need to improve the standard particle swarm optimization algorithm.

The principle of the standard particle swarm algorithm is as follows, in the process of the algorithm, the particle updates its speed and position according to the following equation:

(13) $$\left\{ \begin{array}{l} v_{id}(t+1)=wv_{id}(t)+c_{1}r_{1}(p_{id}(t)-x_{id}(t))+c_{2}r_{2}(p_{qd}(t)-x_{id}(t))\\ x_{id}(t+1)=x_{id}(t)+v_{id}(t+1) \end{array}\right.,$$

where $v_{id}$ is the *d*th velocity component of the *i*th particle; $x_{id}$ is the *d*th position component of the *i*th particle; $P_{id}$ is the optimal position component of the *i*th particle; $P_{qd}$ is the optimal position component of all particles; $c_{1}$ and $c_{2}$ are learning factors, $r_{1}$ and $r_{2}$ are random numbers in [0,1], and ω is an inertia factor. To balance the global detection and local mining capabilities, ω can be dynamically adjusted during the search process. Eberhart et al. proposed a ω linear decreasing adjustment strategy in [45]:

(14) $$\omega =\omega_{\max}-(\omega_{\max}-\omega_{\min})t/T_{f},$$

where $\omega_{\max}$ and $\omega_{\min}$ are the initial and extinction values of the inertia factor, respectively, *t* is the current iteration time, and $T_{f}$ is the number of final iterations. This strategy improves the performance of the algorithm to some extent, but in the initial iteration, ω easily becomes too large and causes oscillation, which leads to low efficiency of the algorithm search. In the later iteration, ω easily becomes too small, leading to lower search accuracy.

To solve this problem, we propose a volatility factor σ which gradually decreases with the number of iterations. The specific equation is as follows:

(15) $$\omega =\omega_{\max}-(\omega_{\max}-\omega_{\min})t/T_{f}+\sigma \times randn,$$

(16) $$\sigma =e^{\left(-t/T_{f}\right)}/2,$$

where randn is a random number obeying a Gaussian distribution with a mean of 0 and a variance of 1. In the initial iteration, ω with large fluctuation factor improves the global detection of the algorithm; in the later iteration, ω with smaller fluctuation factor enhances the local exploitation ability of the algorithm. In general, when ω is attenuated from 0.9 to 0.4, the algorithm works better [46].

**Figure 2:**
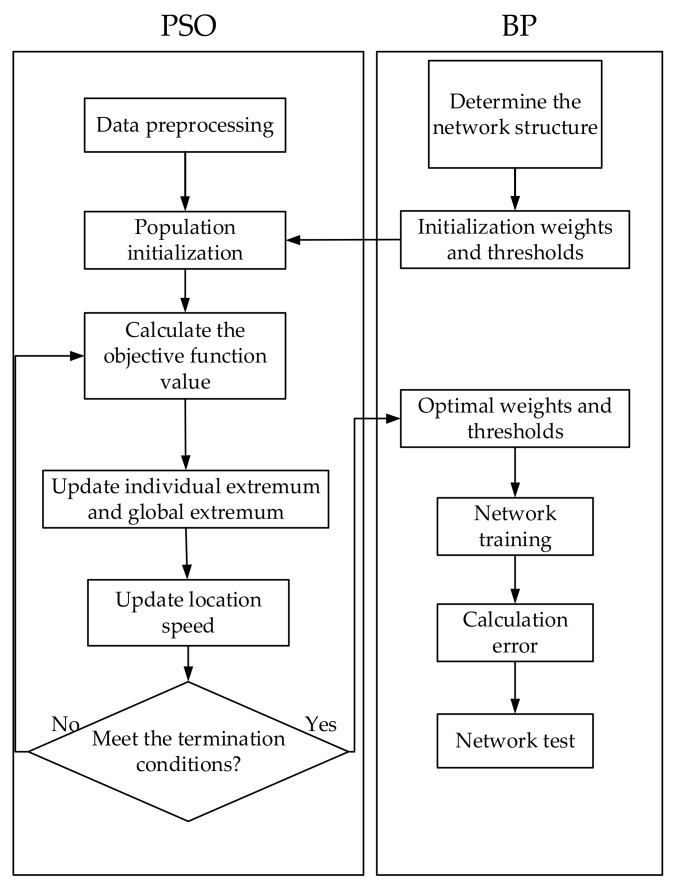
Flowchart of PSO-BP algorithm.

Figure 2 is the flowchart diagram, which shows the steps to optimize the BP algorithm by the improved particle swarm optimization algorithm. 

The specific algorithm steps are as follows:

(1) Particle initialization;
(2) Calculate the value of fitness function;
(3) Find the optimal values of individuals and groups;
(4) Update the speed and position of particles. If the set maximum number of iterations is reached in advance during the optimization process, it stops and outputs the optimal solution at this time. Otherwise, turn to the second step and continue to execute the loop;
(5) Obtain the optimal weight and threshold and assign it to the BP network;
(6) Calculate the error of the BP network model. If the error does not reach the target value, continue to update the weight and threshold of the network until the condition is met.

#### 4.2.3. Hybrid Interpolation Optimization Algorithm Steps

Compared with the ordinary Kriging interpolation and BP neural network interpolation, the PSO-BP-Kriging interpolation algorithm proposed in this paper has higher precision and credibility, and can effectively overcome the shortcomings of using two traditional methods for interpolation estimation. The specific algorithm steps are as follows:

(1) Calculate γ(h) by using sample data and select the appropriate model to fit it;

(2) Determine the network structure. Including learning factors, learning rates, target errors, and maximum number of iterations;
(3) Calculating the variogram $\gamma (h_{k})$ in Equation (12) according to the separation distance $h_{k}$ and the corresponding γ(h) of different sensor node pairs;

(4) Use the PSO algorithm to determine the initial weight of the neural network;
(5) Calculate the network output according to Equation (7);
(6) Update the next iteration weight according to the equation $\Delta \omega_{i,j}^{k,k-1}(n+1)=\partial \Delta \omega_{i,j}^{k,k-1}(n)+\eta \delta_{i}^{k}\mu_{j}^{k-1}$ (∂ is the learning factor and η is the learning rate coefficient);
(7) Use the error between the network output and the sample, and $\gamma^{*}(h_{k})$, to calculate F from Equation (12);

(8) If F≤ε, the weight at this time is the last weight of the network, otherwise, turn to step (4);

(9) Select other samples to test the fitting performance of the network. If the conditions are met, proceed to the next step, otherwise turn to step (4);
(10) Interpolation estimation is performed by using a trained network.

### 4.3. 5G Mobile Communication Network Coverage Area Situation Generation

The coverage situation of 5G mobile communication network is obtained by data collection, data fusion, situational plotting, map support, and other key technologies [47]. Since the focus of this paper is on data acquisition, interpolation estimation, and coverage situation generation, other key technologies are not described in detail here.

Combining the estimated data of the interpolation points in the target area with the data collected by the sensor nodes, the signal strength of each position in the signal coverage area can be represented, and an equal signal strength line of the signal coverage area of the 5G mobile communication network is generated. According to the generated equal signal strength line, the coverage situation of the target area can be obtained, which more intuitively reflects the coverage situation of the real signal in the target area.

## 5. Simulation Experiment Analysis

### 5.1. Simulation Environment Construction

**Figure 3:**
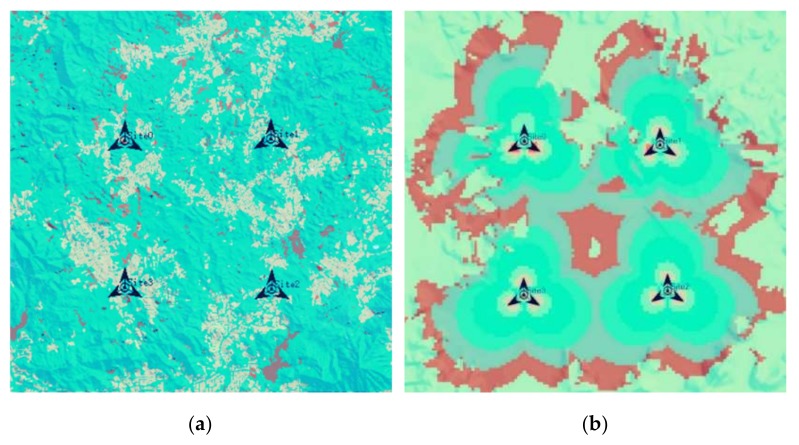
Simulation environment. (**a**) 5G communication test network; (**b**) 5G communication network signal coverage.

In order to verify the performance of the proposed detection technology, the paper uses 5G communication test network as an example to carry out simulation experiments. A 400 m×400 m test network of the 5G communication network deployment company was selected as the actual environment for simulation experiments. There are four 5G communication base stations in the area, as shown in Figure 3a. According to the key data provided by the tester, the signal coverage is obtained by ATOLL simulation as shown in Figure 3b. The simulation parameter settings are shown in Table 1. It is assumed that the number of base stations, location, and signal coverage are unknown when performing simulation experiments. Forty-two sensor nodes were deployed by using a random delivery approach.

In order to facilitate the comparative analysis of interpolation precision, 36 sensor nodes are randomly selected as sampling points, and the remaining 6 points are used as verification points. At the same time, in order to verify the performance of the proposed algorithm, this paper designs several simulation experiments from three aspects: predictive model performance analysis, interpolation optimization algorithm performance analysis and target region coverage situation generation.

### 5.2. Predictive Model Performance Analysis

#### 5.2.1. Predictive Model Accuracy Comparison

**Figure 4:**
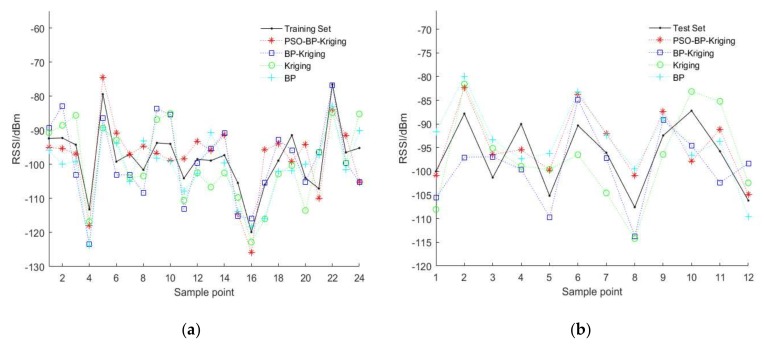
Prediction results. (**a**) Training set prediction results; (**b**) Test set prediction results.

As the core of the algorithm, the prediction accuracy of the model determines the accuracy of the interpolation results. The RSSI values prediction is performed by using PSO-BP-Kriging model, BP-Kriging model, ordinary Kriging model and BP model respectively. Take 36 sampling points selected in Section 5.1 as samples, randomly select 70% of the data for the training set, and the remaining 30% for the test set. The prediction results are shown in Figure 4.

In order to compare the performance of the algorithm, the root mean square error (RMSE) of each model in the training set, and the test set is calculated separately. In the training set, the PSO-BP-Kriging model is 6.178, the BP-Kriging model is 6.829, the ordinary Kriging model is 6.989, and the BP model is 6.866. In the test set, the PSO-BP-Kriging model is 5.993, the BP-Kriging model is 6.513, the ordinary Kriging model is 6.925, and the BP model is 6.871. By comparison, the PSO-BP-Kriging model has the lowest RMSE and the highest accuracy.

In order to make the prediction accuracy of the model more persuasive, 5000 random independent extractions were performed on 36 sensor nodes, and the average RMSE and the average determination coefficient *R*^2^ were calculated. The prediction results of each model are shown in Table 2.

It can be seen from Table 2 that the RMSE of the prediction results of the algorithm in this paper is lower than other models and the *R*^2^ is higher, so the prediction accuracy of our algorithm is higher.

#### 5.2.2. Algorithm Suitability Analysis

In order to verify the applicability of the algorithm, its robustness and complexity are analyzed. First, analyze the robustness of the algorithm. When the sensor node is deployed in the target area for a period of time, due to various reasons, such as energy consumption of nodes, the number of effective sensor nodes and the amount of collected data may be reduced, which inevitably requires the algorithm to be robust. 

**Figure 5:**
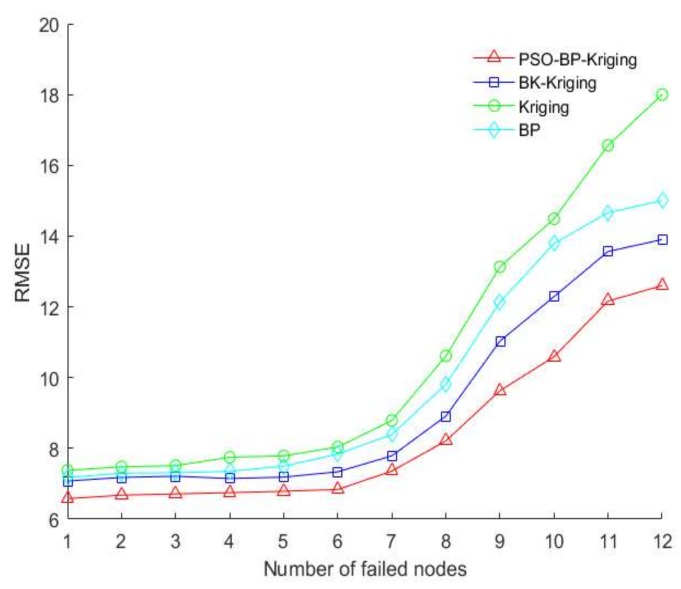
Relationship between RMSE and number of failed nodes in different algorithms.

Therefore, by sequentially increasing the number of failed sensor nodes, the RSSI values are predicted by the above four algorithms, and the predicted results are compared with the original data to calculate RMSE. The comparison results of each algorithm are shown in Figure 5.

It can be seen from Figure 4 that when the number of failed nodes is small, the RMSE variation of each algorithm is relatively stable, and the PSO-BP-Kriging algorithm is the lowest. When the number of failed nodes is large, the RMSE of each algorithm increases, and especially the ordinary Kriging algorithm has a faster growth trend. Therefore, through the above comparative analysis, the prediction result of PSO-BP-Kriging algorithm is more stable and robust.

Then, analyze the complexity of the algorithm. The hybrid interpolation method proposed in this paper, combined with the machine learning method, increases the time complexity of the calculation to a certain extent. However, when restoring the true coverage of a certain area, due to the higher prediction accuracy, usually only less sensor node data is needed. Other algorithms may require multiple acquisitions of sensor node data which, in turn, increases the computational overhead.

In summary, the PSO-BP-Kriging algorithm has the advantages of high precision and good robustness. Considering the three factors of prediction accuracy, robustness, and computational complexity, we can use the algorithm proposed in this paper for prediction when the sensor nodes have certain data processing capability.

### 5.3. Performance Analysis of Interpolation Optimization Algorithm

**Figure 6:**
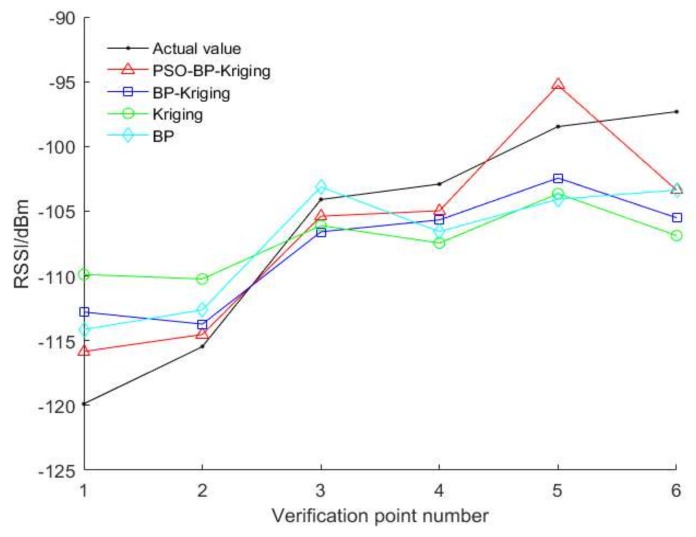
Comparison of interpolation algorithms.

In order to verify the accuracy of the algorithm, we take the six verification points selected in Section 5.1 as an example. The above four algorithms are used to estimate the RSSI values of six interpolation points. The result of the interpolation and the actual value are shown in Figure 6.

The RMSE of the four algorithms is calculated separately, wherein the BP-Kriging interpolation method is 5.0006, the ordinary Kriging interpolation method is 6.7097, the BP model is 5.8327, and the PSO-BP-Kriging is 3.4144. It is not difficult to find that the RMSE of our algorithm is minimal, so the interpolation precision is the highest, and the performance is the best.

### 5.4. Coverage Situation Generation of 5G Network

**Figure 7:**
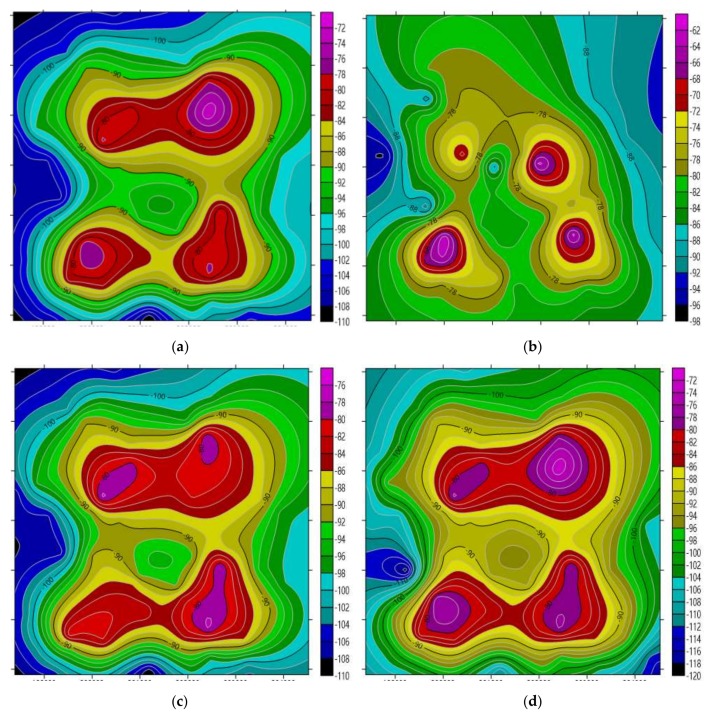
Coverage situation. (**a**) Kriging interpolation results; (**b**) BP interpolation results; (**c**) BP-Kriging interpolation results; (**d**) PSO-BP-Kriging interpolation results.

Interpolation estimation is performed by using the above four algorithms respectively, and the target area coverage situation of the four algorithms can be generated by combining the sensor node acquisition data and the interpolation point estimation data, as shown in Figure 7.

At the same time, 100 position points are randomly selected from the target area. In order to ensure the confidence of the comparison, 5000 sets of experiments were randomly selected to calculate the average RMSE of the interpolation results of the above four algorithms at the 100 position points. Among them, the average RMSE of the ordinary Kriging interpolation is 21.2361, the BP interpolation is 19.7344, the BP-Kriging interpolation is 18.6917, and the PSO-BP-Kriging is 15.3178. After comparison, the algorithm proposed in this paper has higher precision, and the obtained network coverage situation is closest to the actual situation, which can better reflect the real signal coverage of the target area.

### 5.5. Algorithm Validity Analysis

In order to verify the practicability and effectiveness of the algorithm, nine position points in the actual test network of 5G communication in Section 5.1 are randomly selected as test points, and the RSSI values are detected by the traditional road test method. Furthermore, the interpolation estimation values of the nine position points are extracted from the equal signal strength lines generated by the algorithm in this paper. The test results of the two methods are shown in Table 3.

It can be seen from Table 3 that in the area that can be detected by the traditional road test method, the maximum absolute error value of the interpolation result and the road test result is 4.866, and the average absolute error is only 2.156. Therefore, the effectiveness of the algorithm in this paper can be explained. Considering that there are unreachable areas in the traditional road test, the algorithm in this paper can detect a wider range and be more practical.

## 6. Conclusions

Starting from the wireless sensor network (WSN), this paper proposes a new 5G mobile communication network coverage detection technology, which overcomes the limitations of the conventional mobile communication network coverage detection that are time-consuming, labor-intensive, and easily affected by the environment and terrain. Through the improved hybrid interpolation algorithm, the coverage situation generation of the target area of the 5G mobile communication network is realized. The generated coverage of the 5G mobile communication network can intuitively reflect the coverage of the target area, and has certain reference value for measuring the coverage performance of 5G mobile communication networks. Finally, the effectiveness of the generated network coverage situation is verified by simulation experiments. The algorithm proposed in this paper can generate the coverage situation of 5G mobile communication network in the presence of the perceptual blind zone in WSN, and the requirements for wireless sensor nodes are low. It can better meet the operator’s special requirements of 5G mobile communication network coverage in all directions, all weather, with good repeatability, etc. The algorithm is universal and can be widely used in telecommunications, radio committees, and military fields, where there is a large demand for wireless communication network coverage detection. In the future, we will conduct further research on WiMAX network coverage, IoT coverage, sensor network coverage, GSM/CDMA/WCDMA/LTE network coverage, and self-organizing network work in mountainous and remote mining areas.

## Figures and Tables

**Table 1:** Simulation parameter settings.

Simulation Parameters	Configuration Value
Target area size	400 m × 400 m
Path loss model	Okumura-Hata
Standard deviation of shadow fading	10 dB
Carrier frequency	3.4 GHz
Network model	Three sector model
Number of users in each cell	100
Number of sensor nodes	42

**Table 2:** Comparison of different model fitting performance.

Performance Parameter	PSO-BP-Kriging	BP-Kriging	Kriging	BP
RMSE	5.9756	6.5979	6.7193	6.6218
*R* ^2^	0.6541	0.6173	0.6054	0.5946

**Table 3:** Comparison of test results of two methods (unit: dBm).

Method	1	2	3	4	5	6	7	8	9
Road Test	−89.308	−82.852	−103.16	−113.491	−86.475	−108.312	−95.457	−90.887	−79.645
Interpolation	−87.935	−83.674	−105.03	−110.856	−89.317	−107.544	−93.121	−86.021	−81.534

## Appendix A

### Appendix A.1. Table of Abbreviations

**Abbreviation**	**Full Name**
5GNOW	5th Generation Non-Orthogonal Waveforms for Asynchronous Signaling
METIS	Mobile and Wireless Communications Enablers for the Twenty-Twenty Information Society
5G PPP	5G Public-Private Partnership
RSSI	Received Signal Strength Indicator
PSO	Particle Swarm Optimization
BP	Back Propagation
PSO-BP	Particle Swarm Optimization BP Neural Network
BP-Kriging	Kriging-BP Neural Network
PSO-BP-Kriging	Particle Swarm Optimization and Kriging -BP Neural Network
RMSE	root mean square error

### Appendix A.2. Comparison Table of Related Work

**Method**	**Characteristics**	**Difference**
Signal propagation model	Low computational complexity	Low precision, existing models are not applicable
Newton interpolation	High precision	High computational complexity
IDW interpolation	High precision when interpolating points are dispersed	Poor spatial correlation, large amount of calculation
Kriging interpolation	Good spatial correlation and high precision	Spatial local features are masked
BP neural network	High precision	Poor spatial correlation, local convergence
PSO-BP model	Higher precision	Poor spatial correlation
BP-Kriging model	Good spatial correlation and high precision	Local convergence

### Appendix A.3. Mathematical Calculations and Derivations

(1) Kriging interpolation:

First assume that the regionalized variable R(x) satisfies the second stationary assumption, its mathematical expectation is *p*, and the covariance function c(h) and the variogram γ(h) exist:

(A1) $$\begin{array}{l} E[R(x)]=p\\ c(h)=E[R(x)R(x+h)]-p^{2}\\ \gamma (h)=\frac{1}{2}E[R(x)-R(x+h){]}^{2} \end{array}$$

Suppose there are a total of *m* measured points in the neighborhood of the point *x* to be estimated, and the sample value is $R(x_{i})(i=1,2,\cdots ,m)$, then the formula of the Kriging interpolation is:

(A2) $$R^{*}(x)=\sum_{i=1}^{m}\lambda_{i}R(x_{i}),$$

where $\lambda_{i}$ is a weighting coefficient, indicating the degree of contribution of the observed value $R(x_{i})$ at each spatial sample point $x_{i}$ to the estimated value $R^{*}(x)$. It can be seen that the key to Kriging interpolation is to calculate the weight coefficient $\lambda_{i}$. To do this, the following two conditions must be met:

1. Unbiased. To make $R^{*}(x)$ an unbiased estimator of $R(x_{i})$, then:

(A3) $$E[R^{*}(x)]=E[R(x)].$$

When E[R(x)]=p, that is, when $E[\sum_{i=1}^{m}\lambda_{i}R(x_{i})]=\sum_{i=1}^{m}\lambda_{i}E[R(x_{i})]=p$, then

(A4) $$\sum_{i=1}^{m}\lambda_{i}=1.$$

At this time, $R^{*}(x)$ is an unbiased estimator of $R(x_{i})$.

2. Optimization condition. Under the condition of satisfying unbiasedness, the estimated variance is

(A5) $$\sigma_{E}^{2}=E{\left[R(x)-R^{*}(x)\right]}^{2}=E{\left[R(x)-\sum_{i=1}^{m}\lambda_{i}R(x_{i})\right]}^{2}.$$

Using the covariance function expression, it can be further expressed as

(A6) $$\sigma_{E}^{2}=c(x,x)+\sum_{i=1}^{m}\sum_{j=1}^{m}\lambda_{i}\lambda_{j}c(x_{i},x_{j})-2\sum_{i=1}^{m}\lambda_{i}c(x_{i},x).$$

In order to minimize the estimated variance, according to the Lagrangian multiplier principle, let

(17) $$F=\sigma_{E}^{2}-2\mu (\sum_{i=1}^{m}\lambda_{i}-1{)}_{.}$$

Find the partial derivative of *F* for $\lambda_{i}$ and μ, and let it be 0, then get the Kriging equations:

(18) $$\left\{ \begin{array}{l} \frac{\partial F}{\partial \lambda_{i}}=2\sum_{j=1}^{m}\lambda_{j}c(x_{i},x_{j})-2c(x_{i},x)-2\mu =0\\ \frac{\partial F}{\partial \mu }=-2(\sum_{i=1}^{m}\lambda_{i}-1)=0 \end{array}\right..$$

Hence,

(19) $$\left\{ \begin{array}{l} \sum_{j=1}^{m}\lambda_{j}c(x_{i},x_{j})-\mu =c(x_{i},x)\\ \sum_{i=1}^{m}\lambda_{i}=1 \end{array}\right..$$

Solve the Equation (A9), find the weight coefficient $\lambda_{i}$ and the Lagrangian coefficient μ, and substitute Equation (A5) to obtain the Kriging estimated variance:

(20) $$\sigma_{E}^{2}=c(x,x)-\sum_{i=1}^{m}\lambda_{i}c(x_{i},x)+\mu .$$

In the presence of the variogram, according to the relationship between the covariance and the variogram, γ(h)=c(0)−c(h), the variogram can also be used to represent the Kriging equations and the estimated variance:

(21) $$\left\{ \begin{array}{l} \sum_{i=1}^{m}\lambda_{i}\gamma (x_{i},x_{j})+\mu =\gamma (x_{i},x)\\ \sum_{i=1}^{m}\lambda_{i}=1 \end{array}\right..$$

Solve the Equation (A9), find the weight coefficient $\lambda_{i}$ and the Lagrangian coefficient μ, and substitute the Equation (A5) to obtain the Kriging estimated variance:

(22) $$\sigma_{K}^{2}=\sum_{i=1}^{m}\lambda_{i}\gamma (x_{i},x)-\gamma (x,x)+\mu .$$

(2) BP neural network:

The node function of the BP neural network is

(23) $$u_{i}^{k}=f(\sum_{j=1}^{l_{k-1}}w_{i,j}^{k,k-1}u_{j}^{k-1}-\theta_{i}),$$

where $u_{j}^{k-1}$ is the output of the *j*-th node of the k−1th layer, $u_{i}^{k}$ is the *i*-th node output of the *k*-th layer, $w_{i,j}^{k,k-1}$ is the input weight of the *j*-th node from the k−1 th node to the *i*-th node of the *k*-th layer, $\sum_{j=1}^{l_{k-1}}w_{i,j}^{k,k-1}u_{j}^{k-1}-\theta_{i}$ is the net input of the *i*-th node, $\theta_{i}$ is the threshold of the node, f is a nonlinear action function, usually taking the sigmoid function f(x)=1/(1+exp(−x)); for the input layer, there is $u_{i}=f(x_{i}-\theta_{i})$.

The iterative process (called neural learning) used to determine the appropriate weight minimizes the objective function:

(24) $$F=\frac{1}{2}E[\sum_{i=1}^{N}\sum_{j=1}^{M}{\left(y_{ij}-o_{ij}\right)}^{2}],$$

where $o_{ij}$ represents the actual output of the network and $y_{ij}$ represents the expected output, *N* is the number of samples in the training dataset, *M* is the number of nodes in the output layer, F is used as the standard for learning, and learning continues until F is less than the set error ε.

Modify the next iteration weight in reverse according to Equation (A15):

(25) $$\Delta \omega_{i,j}^{k,k-1}(n+1)=\partial \Delta \omega_{i,j}^{k,k-1}(n)+\eta \delta_{i}^{k}\mu_{j}^{k-1},$$

where *n* is the number of iterations, $\delta_{i}^{k}$ is the learning signal of the *i*-th node of the *k*-th layer, ∂ is the learning factor, and η is the learning rate coefficient.

Assuming there are *P* samples, each sample has *N* input components $X=(x_{1},x_{2},\ldots ,x_{N})$ and *M* output components $Y=(y_{1},y_{2},\ldots ,y_{M})$, and the basic steps of an *L*-layer perceptron BP algorithm are:

(1) Determine the hidden layer and the number of nodes per layer;
(2) Determine the network structure;
(3) Input sample data;
(4) Calculate the output value of each layer node according to the node function Equation (A13);
(5) Modify the weight of the n+1 th iteration;

(6) Calculate the error $F=\frac{1}{2}E[\sum_{p=1}^{N}\sum_{j=1}^{M}{\left(y_{j,p}-o_{j,p}\right)}^{2}]$ of the network output value and the expected output value, where $y_{j,p}$ is the expected value of the output of the *j*-th component of the *p*-th sample, and $o_{j,p}$ is the network output value, and when the objective function F value is less than the given allowable error ε, the learning process ends, completing the training process of the network, otherwise return to step (3);

(7) Select different samples from the training set to detect the generalization ability of the network. If the error between the network output value and the actual value of the detected sample is within the allowable range, the network training quality meets the requirements. Otherwise, re-adjust the parameters and repeat the above network training until the network training error meets the requirements.
